# Application of Family Stress Theory: Predicting Wellbeing by Demands, Resources and Perceptions Among Caregivers of Older Adults

**DOI:** 10.1111/famp.13100

**Published:** 2025-01-21

**Authors:** Jacky C. P. Choy, Terry Y. S. Lum, Doris S. F. Yu, Gloria H. Y. Wong

**Affiliations:** ^1^ Department of Social Work and Social Administration The University of Hong Kong Hong Kong Hong Kong; ^2^ Sau Po Centre on Aging The University of Hong Kong Hong Kong Hong Kong; ^3^ School of Nursing The University of Hong Kong Hong Kong Hong Kong; ^4^ School of Psychology and Clinical Language Sciences University of Reading Reading UK

**Keywords:** additional caregiving roles, care burden, caregiving for older adults, family stress theory, multiple family caregivers

## Abstract

Family caregivers of older adults are at risk of high care burden and reduced quality of life. Existing research and practices had primarily focused on the caregiving dyad. However, it is often observed that multiple family members are involved in caregiving for older adults. We applied family stress theory to understand family caregiving and examined how care demands, resources, and perceptions are associated with and predict caregiver well‐being. Participants in this study were low‐income family caregivers who received caregiver allowance and provided care for an older adult with care needs in the community in Hong Kong. Two waves of data, including baseline data from 358 caregivers and 2‐year follow‐up data from a subsample of 93 caregivers, were collected. We used hierarchical regression to predict care burden and quality of life at baseline and follow‐up, respectively, by care demands, resources, and perceptions after controlling for the context of care. Results show that additional caregiving roles, quality of relationship with the older adult, and satisfaction with family support were associated with care burden and quality of life at baseline. Predictors of lower care burden at 2‐year follow‐up were discontinuation of additional caregiving roles, increase in size of caregiving family, and the use of domestic helper. Applying family stress theory to understand the caregiving process reveals the significance of additional caregiving roles, the involvement of multiple caregivers, and caregivers' perceptions about family support in enhancing caregiver well‐being, underscoring the need to focus on these factors when designing and implementing caregiver support services.

1

Family plays an essential role in our societies for taking care of older adults. Caring for an older adult with debilitating conditions, such as cancer, diabetes, dementia, and other chronic conditions causing frailty, is a stressful role (Kasper et al. 2015) that is associated with a high level of care burden and poorer quality of life (Kim and Schulz 2008). Moreover, family caregivers even had increased risks for depression and premature mortality (Carretero et al. 2009; Schulz and Beach 1999). To protect them and to sustain this important community asset of informal care, caregiving research has been conducted to examine the mechanism of caregiver stress. Most previous research primarily focused on the caregiving dyad, consisting of one caregiver and one care recipient, and individual‐ and dyadic‐level factors in their conceptualization of caregiver well‐being (Brodaty and Donkin 2009; Chiao, Wu, and Hsiao 2015; Gilhooly et al. 2016). However, it had been observed that family care for older adults is indeed shared among multiple family members (Harvath et al. 2020). In a recent cohort study of 1871 family caregivers of people living with dementia in the U.S. (Lai et al. 2022), 63% reported that they were sharing the caregiving role with other informal caregivers, with lower odds of experiencing negative emotional burden compared with caregivers providing care alone. To advance our understanding of family caregiving and better support the caregivers, it is imperative to conceptualize family care as a role faced by the whole family unit and take family‐level factors into consideration for understanding the caregiving process and caregiver well‐being. In this study, we apply the family stress theory to understand family caregiving as a process and examine how care demands, resources, and perceptions associate with and predict well‐being among family caregivers of older adults in Hong Kong. We use care burden and quality of life as indicators of caregiver well‐being to reflect the positive and negative dimensions of well‐being respectively (Cunningham, Cunningham, and Roberston 2019).

### 1.1 Dyadic Conceptualization of Caregiving and Its Limitations

Family caregiving has largely been conceptualized as a stress and coping process (Pearlin et al. 1990) and mainly focused on the caregiving dyad, consisting of the care recipient and a primary caregiver. Risk factors of caregiver wellbeing have been categorized into (a) patient factors, (b) caregiver factors, and/or (c) dyadic factors (Brodaty and Donkin 2009; Chiao, Wu, and Hsiao 2015; Gilhooly et al. 2016). Examples of patient factors included disease severity, functional impairment, and behavioral and psychological symptoms; caregiver factors consisted of financial resources, coping skills, and perception of the caregiving role; and dyadic factors referred to the type of relationship (e.g., spousal/non‐spousal), co‐residence, and quality of relationship within the dyad. Moreover, the caregiving dyad is often assumed to be a static dyadic relationship (Schulz et al. 2020). Following this major conceptualization, a wide range of caregiver intervention and support services have been developed to improve caregiver well‐being. The major intervention mechanism was to increase the capacity of the primary caregiver to cope with the demands arising from the care recipient, covering their inner thoughts, emotions, and/or the care task itself. Yet, the effect of caregiver intervention in general was found to be small and, at best, moderate (Schulz et al. 2020). These findings suggested that we might have overlooked some other potential contributors to caregiver well‐being, and thus needed to rethink family caregiving.

### 1.2 Involvement of Multiple Family Members in Caregiving

It has been continuously observed that family care is shared among multiple family members (Harvath et al. 2020). In the 1990s, Keith (1995) identified three types of family caregiving systems, namely, *primary*, *partnership*, and *team* and suggested that family care was shared by multiple family members with family size and gender composition as potential factors determining the type of system. Afterwards, however, not much caregiving research addressed family as a holistic unit and shared family care by multiple caregivers. In a recent cohort study of 1871 family caregivers of people with dementia in the U.S., Lai et al. (2022) found that 63% were sharing the caregiving role with other informal caregivers, with lower odds of experiencing negative emotional burden compared with caregivers who provided care alone. These recent findings further supported that family caregiving is not a matter for a single caregiver.

The involvement of other family members was mostly addressed as *social/family support* towards the primary caregiver (McCabe, You, and Tatangelo 2016) and known as a protective factor for well‐being (Brodaty and Donkin 2009). Such conceptualization may overlook the possibility of other family members as active caregivers and the support provided by the primary caregiver towards other family members. For instance, it was reported that caregivers had additional caregiving roles, also known as *compound caregiving*, in the family that they looked after of their child/grandchild or other family members with special needs simultaneously (Carr and Utz 2020; Grossman and Webb 2016; Perkins 2010). These findings provided the grounds to look beyond the dyadic caregiving relationship and take the whole family into perspective in the context of family caregiving for older adults.

### 1.3 Theoretical Framework: Family Stress Theory

Family stress theory (Hill 1949) suggests that families continue to experience periodic and acute stressors, and their responses determine how some families adapt and adjust to stress and some fail. Building on Hill's work and his ABCX model (1949), others further advanced the theory by developing other family stress models, including the Family Adjustment and Adaptation Response model (Patterson 1988) and the Contextual Model of Family Stress (Boss, Bryant, and Mancini 2016). They altogether offered a perspective that adjustment and adaptation to stress (or not) is a result of the interplay among stressors faced by the family, resources available for the stressors, and perceptions about the stressors and resources. To maintain or restore family well‐being, families attempt to balance their demands and resources. And the relevant perceptions influence how well the family can make use of their resources to cope with the demands.

Family caregiving for an older adult with care needs is an enduring journey, along with changes in the condition of the older adult and in the roles of individual family members. Guided by the family stress theory, care needs of the older adults and additional caregiving roles can be seen as care demands, and care‐related resources available to the caregivers, such as support from other family members and formal services, are resources for coping with this stressor. Perceptions, including cognitive perceptions, such as how they think about the caregiving role, and affective perceptions, such as how they feel about other family members, would affect the coping response (Boss, Bryant, and Mancini 2016). Moreover, when caregiving conditions change, there might be a pile‐up or accumulation of care demands (e.g., decline in older adult's daily functioning); families would need to increase/redistribute their resources (e.g., involving more members in caregiving, re‐negotiating the care arrangement within the family; Patterson 1988) along the caregiving journey to maintain a balanced family functioning. A recent systematic review on the application of family stress theory (Casaburo et al. 2023) revealed that there were so far no previous studies that directly applied family stress theory to understand family caregiving for older adults.

By applying family stress theory to the context of caregiving, the current study challenges the predominant stress and coping conceptualization that primarily focuses on the caregiving dyad. We aim to take the whole family into perspective by capturing both care demands and resources at the family level and examining how the family‐level factors affect caregiver well‐being. Moreover, while previous caregiving research often assumed a static relationship between the caregiver and the care recipient, caregiving is a dynamic process in which the care needs of older adults and the involvement of caregivers may fluctuate. Family stress theory will guide us in conceptualizing and differentiating the accumulation of demands, redistribution of resources, and changing perceptions, as well as their impacts on caregiver well‐being.

### 1.4 Context of Current Study: Family Caregiving for Older Adults in Hong Kong

Hong Kong is a high‐income Chinese society that has undergone rapid changes in family systems (Lum and Chow 2008). While there is a contemporary shift in filial behaviors from an authoritarian obligation to a more egalitarian and utilitarian form (Lum et al. 2016b; Yeh et al. 2013), family caregiving for older adults with care needs is still largely considered as an encouraged family responsibility. Older adults in need of care expressed a strong preference for aginginplace, with over 80% of lower‐income older persons preferring to stay home even when they become dependent (Lum et al. 2016a). For family caregivers, the actual practice of caregiving has become increasingly symbolic (Ting 2009) or involves financial support over direct care provision (Cheung and Kwan 2009). In addition, it is common for families in Hong Kong to hire a live‐in foreign domestic helper for housekeeping and caring for young children or older adults with care needs. Nearly 10% of households employ a foreign domestic helper, accounting for almost 5% of the city's population (Choy et al. 2022). This practice is affordable for many local families, as the minimum monthly salary for foreign domestic helpers is HKD 4990 (~USD 640; The Government of HKSAR 2024), while the median monthly domestic household income is HKD 35,000 (~USD 4487) for households of three and HKD 47,700 (~USD 6115) for households of four (Census and Statistics Department of HKSAR Government 2024). For taking care of older adults, in particular, 21.3% of older adults with care needs in the community identified foreign domestic helpers as their primary caregivers (Census and Statistics Department of HKSAR Government 2023), and between 26% and 54% of people living with dementia received care from them. (Choy et al. 2022). Local studies showed that the use of domestic helpers moderates the relationship between stressors (care needs) and caregiver distress in spousal (Chong et al. 2014) and adult child caregivers (Chong et al. 2017). However, its impact together with the involvement of multiple family caregivers on caregivers' well‐being is yet to be explored.

As aging societies, including Hong Kong and other developed cities around the world, are looking for viable and sustainable ways to support family caregivers of older adults, understanding how the family as a unit coped with the caregiving role and how family‐level factors impact a caregiver's wellbeing is needed, particularly relevant to the global trends of decreasing fertility rates and reducing size of extended families.

### 1.5 Research Objectives

In the present study, we applied the family stress theory to understand the caregiving process among caregivers who take care of an older adult in the community in Hong Kong. We conceptualized the care needs of older adults and additional caregiving roles as care demands, formal and informal support from the family and community as resources, and quality of relationship with the older adults and satisfaction towards family support as perceptions towards care demand and resource, respectively.

The objective of this study is two‐fold: First, we examine the relationship of care demands, resources, and perceptions with well‐being of caregivers after controlling for the context of care. We hypothesized that higher care demands (impairment in activities of daily living (ADLs), impairment in instrumental activities of daily living (IADLs), and additional caregiving role) would be associated with higher care burden and poorer quality of life; more resources (size of caregiving family, use of domestic helper, and use of caregiver support service) and better perceptions (quality of relationship with older adult and satisfaction with family support) would be associated with lower care burden and better quality of life. In addition, we hypothesized that family‐level factors, including additional caregiving role, size of caregiving family, and satisfaction with family support, would be associated with care burden. Second, taking the pile‐up of stressors and the adaptive responses of a family along the caregiving process into consideration, we aimed to identify the predictors of caregiver well‐being among factors of care demands, resources, perceptions, and their changes.

## 2 Method

### 2.1 Data Source and Participants

This was a secondary data analysis of a two‐year longitudinal research study conducted on caregivers who enrolled in the Pilot Scheme on Living Allowance for Carers of Elderly Persons from Low‐Income Families provided by the Hong Kong government between 2015 and 2017. The original research aimed to evaluate the impact of this caregiver allowance program. Under this program, caregivers would receive a cash allowance of HKD 2000 monthly (~USD 255) and regular caregiver support service from the district community center. Eligible caregivers were (a) taking care of an older adult in need of and waiting for long‐term care services as determined by a comprehensive long‐term care needs assessment; (b) from a low‐income family with household income below 75% of the local median monthly domestic household income and not receiving other forms of social security; and (c) identified as the primary caregiver and provided at least 80 h of care to the older person per month.

Two waves of data collection were conducted in the original research. The first wave consisted of 358 family caregivers who completed a questionnaire about their characteristics and caregiving profile. Two years later, 211 participants continued to enroll in the caregiver allowance program and to take care of the older adults in the community after excluding those who ceased care provision, older adults who passed away, or moved into a residential care home. Among them, a subsample of 93 caregivers participated in the second wave of data collection in fulfillment of the requirement for the evaluation study. The first wave of data from 358 participants and the second wave from 93 participants constituted the baseline and follow‐up data, respectively, in the current study.

### 2.2 Procedures

At baseline, participants were randomly selected from a pool of caregivers who were enrolling in the caregiver allowance program. At the 2‐year follow‐up, we recruited a consecutive subsample based on the sequence of the start date of receiving the allowance. Trained research assistants contacted and invited participants to the research by phone. Face‐to‐face interviews were then conducted at the participant's home or in a quiet location convenient to the participant. Written informed consent was obtained from participants prior to the interviews. The research was approved by the Human Research Ethics Committee of The University of Hong Kong (Reference No.: EA1502008 & EA1704021).

### 2.3 Measures

#### 2.3.1 Caregiver Wellbeing

##### 2.3.1.1 Care Burden

We measured the care burden of the caregiver as an indicator of well‐being and used the Chinese version of the 22‐item Zarit Burden Interview (ZBI; Chan, Lam, and Chiu 2005). ZBI is a widely adopted and validated measure for care burden among caregivers of older persons across cultures (Adelman et al. 2014). Participants were asked to rate how often they felt the distress described in each item (e.g., Do you feel stressed between caring for your relative and trying to meet other responsibilities for your family or work?) Are you afraid of what the future holds for your relative? Do you feel strained when you are around your relative? on a 5‐point Likert scale from (0) *never* to (4) *always*. The item scores were summed to obtain a total score ranging from 0 to 88, with a higher score indicating a higher level of care burden. The instrument had an excellent level of internal consistency among the current sample (Cronbach's alpha = 0.92).

##### 2.3.1.2 Quality of Life

We included the quality of life of caregivers as a second indicator of caregiver well‐being in order to reflect the positive aspect of well‐being (Cunningham, Cunningham, and Roberston 2019). It was measured by the European Health Interview Survey—Quality of Life (EUROHIS‐QOL) 8‐item index (Schmidt, Mühlan, and Power 2006). Participants were asked rate how satisfied they were in eight different aspects of their life (e.g., How satisfied are you with your health? Do you have enough energy for everyday life? How satisfied are you with the conditions of your living place?) on a 5‐point Likert scale from (1) *not at all* to (5) *completely*. Items were translated into Chinese for administration. The average score of the items, which ranged from 1 to 5, was used to represent the overall quality of life, with a higher score indicating better quality of life. The instrument demonstrated a good level of internal consistency among the current sample (Cronbach's alpha = 0.81).

#### 2.3.2 Care Demands

##### 2.3.2.1 Activities of Daily Living

We assessed the functional abilities of the older adults to understand their care needs. We measured ADLs by the Barthel Index (Collin et al. 1988), in which participants were asked to report on the older adult's actual performance in 10 ADLs. For the mobility and transfer items, the possible ratings were (0) unable, (5) requiring a wheelchair or major assistance, (10) needing minor help from others, and (15) independent. For bathing and grooming, the ratings were (0) dependent and (5) independent. For the remaining six ADLs, the ratings were (0) unable or dependent, (5) needing some help, and (10) independent. The Barthel Index is extensively validated for functional disability of older persons and validated in Hong Kong (Hartigan 2007; Leung, Chan, and Shah 2007). The total score ranged from 0 to 100, with a higher score indicating a higher functional level. It demonstrated a good level of internal consistency among the current sample (Cronbach's alpha = 0.89).

##### 2.3.2.2 Instrumental Activities of Daily Living

We also measured IADLs of the older person using the Hong Kong Chinese version of the Lawton IADLs Scale, a reliable and valid measure locally tested among older persons in Hong Kong (Tong and Man 2002). For each of the 9 IADLs, such as transportation, shopping, and meal preparation, participants described the highest capabilities of the older person as (0) dependent, (1) needing some help, or (2) independent. The total score ranged from 0 to 18, with a higher score reflecting a higher level of functioning. It had a good level of internal consistency among the current sample (Cronbach's alpha = 0.87).

##### 2.3.2.3 Additional Caregiving Role

To capture their care demands at the family level, participants were asked to report if they needed to take care of other family members (*yes*/*no*), including another older adult with care needs, a child aged under 12, and a relative with a physical disability, intellectual disability, or severe mental illness.

#### 2.3.3 Resources

##### 2.3.3.1 Size of Caregiving Family

The size of the caregiving family directly represented the allocated manpower in care at the family level and indirectly reflected an aggregate of knowledge and skills for care contributed by individual members. To measure the size of the caregiving family, we asked the participants to report the family members (including self) involved in four aspects of care, namely ADL support, IADL support, emotional support (for the older person), and major decision‐making. We then computed the size of the caregiving family by summing up the number of members involved in each aspect of care and removing duplicates.

##### 2.3.3.2 Use of Live‐In Domestic Helper

In Hong Kong, it is common to hire a foreign domestic helper to take care of older adults (Choy et al. 2022). The foreign domestic helpers usually live with the older adults in need of care, directly take care of their daily living, and support the family caregivers on household chores and caregiving duties. The cost of hiring the help could be afforded by the primary caregiver alone or shared among the family members. Participants reported whether they hired a live‐in domestic helper to assist in caregiving in the household of the older person (*yes*/*no*).

##### 2.3.3.3 Use of Caregiver Support Service

Caregiver support service in Hong Kong is mostly provided by non‐governmental organizations under government subvention. Family caregivers could receive training, counseling, support groups, service referrals and other psychosocial support at a very low cost. Participants reported whether they participated in any caregiver training program or support group in the last 12 months (*yes*/*no*). This measure represented whether the caregiver received formal support from the community.

#### 2.3.4 Perceptions

##### 2.3.4.1 Quality of Relationship With Care Recipient

Quality of relationship with care recipient (CR; i.e., the older adult) was regarded as the caregiver's perception towards the care demand. We asked the participants to rate their perceived quality of relationship with CR on a 5‐point Likert scale from (1) *very poor* to (5) *very good* by a single item.

##### 2.3.4.2 Satisfaction With Family Support

Satisfaction with family support represented how the caregiver perceived the support he/she received from other family members. We asked the participants to rate their satisfaction with the support provided by other family members on a 5‐point Likert scale from (1) *very dissatisfied* to (5) *very satisfied* by a single item.

#### 2.3.5 Context of Care

Socio‐demographic information of the caregivers and older adults they cared for, including their age, gender, education level, marital status, employment status, and relationship (husband, wife, son, son‐in‐law, daughter, daughter‐in‐law, other relatives/friends), and their care arrangement, including cohabitation with CR (*yes*/*no*) and daily hours of caregiving by the participant, were considered as the context of care. For daily hours of caregiving, we asked the participants to estimate how many hours they spent taking care of the older adults per day on average over the last week, including the provision of ADL support, IADL support, emotional support, and companionship.

### 2.4 Data Analysis

#### 2.4.1 Participant Profile

We provided descriptive statistics of the baseline characteristics to illustrate the profile of the cross‐sectional sample (*N* = 358) and the 2‐year longitudinal follow‐up subsample (*N* = 93). To assess the attrition bias, we used independent *t*‐tests and chi‐squared tests to compare the baseline characteristics of participants who were included in the follow‐up subsample (*n* = 93) with those who were not (*n* = 118). Paired samples *t*‐tests and McNemar's tests were conducted to explore the changes in their demand, resources, perceptions, and wellbeing over time.

#### 2.4.2 Baseline Data

We used hierarchical regression to examine the relationship of care demands, resources, and perceptions with care burden and quality of life separately after controlling for the context of care. The blocks of predictors were entered into the regression model in two steps: first, context of care; and then, care demands, resources, and perceptions. This analytical design aimed to examine the three blocks of predictors at the same level of hierarchy, without exploring their order. We adopted a theory‐driven approach to select factors related to care demands, resources, and perceptions for predicting care burden and quality of life. For selecting factors related to the context of care, we referred to previous studies for known factors of care burden. These variables included caregiver gender, caregiver education level, cohabitating with CR, daily hours of caregiving (i.e., caregiving loads), and monthly household income per head (i.e., financial stress; Adelman et al. 2014; Chiao, Wu, and Hsiao 2015).

#### 2.4.3 Longitudinal Data

We used hierarchical regression to predict care burden and quality of life by care demands, resources, perceptions, and their changes in 2 years after controlling for the baseline score and context of care. Similar to the baseline model, the blocks of predictors were entered into the model in two steps: first, context of care and the baseline score; second, care demands, resources, and perceptions. To reflect the pile‐up of stressors and the adaptive responses of a family along the caregiving process, we computed change scores or categorical changes for care demands, resources, and perceptions. The dependent variable was the follow‐up score of care burden and quality of life, respectively, in two separate analyses. As the sample size (*n* = 93) was insufficient to include all the predictors for a valid analysis, we adopted a combined theory‐ and data‐driven approach to select the predictors. First, to explore their predictive value after controlling for other factors, all the factors (baseline values and their changes) were entered into the regression model after controlling for the contextual factors and baseline care burden or quality of life (model 1 in Table 4). As we aimed to identify the predictors that accounted for the most variance of the dependent variable, we examined the part correlation coefficient of each factor and selected those with at least a small effect size (*r* > 0.01; Warner 2012). These factors were then entered into another regression model after controlling for baseline care burden or quality of life (model 2 in Table 4). All statistical analyses were performed by using the IBM SPSS Statistics package version 28.0.

## 3 Results

### 3.1 Sample Characteristics

Table 1 shows the baseline characteristics of all participants (*N* = 358) and the 2‐year follow‐up subsample (*n* = 93). The majority of the participants were female (70%) who took care of a female older adult (61%) living in the same household (87%). Adult child was the most common type of relationship with the older adults (66%). At baseline, there were one to two family members (1.59 ± 0.87) on average involved in caregiving, with 29% of families hiring a domestic helper to assist in care. They spent 13.3 ± 7.0 h per day taking care of the older adult, with 17% reporting that they needed to take care of other family members as well. For the attrition bias, the 2‐year subsample did not differ from those who were lost to follow‐up, except that they were older (58.5 ± 10.5 vs. 55.6 ± 9.8 years old), *t*(209) = 2.21, *p* = 0.036, and less likely to be employed or self‐employed (33% vs. 48%), χ^2^(1, *N* = 211) = 4.80, *p* = 0.029.

**TABLE 1:** Baseline characteristics of all participants and the 2‐year subsample.

Characteristic (possible range)	All	2‐year subsample	Loss to follow‐up	*t*/*χ* ^2^ test
	(*N* = 358)	(*n* = 93)	(*n* = 118)	
	*M* (SD)/*n* (%)			*p*
**CG demographics**				
Age	57.8 (10.4)	58.5 (10.5)	55.6 (9.8)	0.036*
Gender, female	252 (70%)	69 (74%)	81 (69%)	0.377
Education level				0.733
No formal education	11 (3%)	1 (1%)	4 (3%)	
Primary	77 (22%)	18 (19%)	23 (20%)	
Secondary	216 (60%)	59 (63%)	74 (63%)	
Tertiary or above	54 (15%)	15 (16%)	17 (14%)	
Marital status, currently married	213 (60%)	48 (52%)	63 (53%)	0.797
Employment status, (self‐) employed	144 (40%)	31 (33%)	57 (48%)	0.029*
Relationship with CR				0.573
Husband	11 (3%)	17 (18%)	5 (4%)	
Wife	81 (23%)	5 (5%)	23 (19%)	
Son	94 (26%)	19 (20%)	33 (28%)	
Daughter	143 (40%)	45 (48%)	50 (42%)	
Daughter‐in‐law	24 (7%)	6 (7%)	7 (6%)	
Grandchild	2 (1%)	—	—	
Sibling	1 (0.3%)	—	—	
Friend	2 (1%)	1 (1%)	—	
Cohabitating with CR	310 (87%)	81 (87%)	103 (87%)	0.967
Daily hours of caregiving	13.3 (7.0)	14.2 (6.8)	12.7 (6.8)	0.109
Monthly household income per head^1^	428 (223)	431 (234)	482 (219)	0.409
**CR demographics**				
Age	83.4 (8.7)	83.5 (8.7)	82.2 (8.6)	0.273
Gender, female	219 (61%)	66 (71%)	78 (66%)	0.451
**Care demands**				
Barthel Index for ADLs (0–100)	58.0 (30.3)	60.1 (29.7)	61.8 (28.7)	0.680
Lawton IADLs (0–18)	2.70 (3.84)	2.80 (3.51)	2.92 (4.04)	0.812
Additional caregiving role	60 (17%)	12 (13%)	16 (14%)	0.889
**Resources**				
Size of caregiving family	1.59 (0.87)	1.56 (0.76)	1.54 (0.91)	0.887
Use of live‐in domestic helper	103 (29%)	25 (27%)	32 (27%)	0.969
Use of caregiver support service	41 (12%)	11 (12%)	10 (9%)	0.419
**Perceptions**				
Quality of relationship with CR (1–5)	3.78 (0.79)	3.74 (0.75)	3.93 (0.77)	0.073
Satisfaction with family support (1–5)	3.27 (0.81)	3.25 (0.83)	3.27 (0.82)	0.835
**Caregiver wellbeing**				
Zarit Burden Interview (0–88)	31.2 (16.2)	31.0 (15.0)	31.4 (16.9)	0.854
EUROHIS‐QoL 8‐item index (1–5)	3.13 (0.52)	3.11 (0.50)	3.14 (0.53)	0.751

*Note:* Currency was converted from Hong Kong dollar to US dollar with the rate of 1 USD, 7.8 HKD. *p < 0.05.

Abbreviations: ADLs, Activities of daily living; CG, Caregiver; CR, Care recipient; EUROHIS‐QoL, European Health Interview Survey – Quality of Life; IADLs, Instrumental activities of daily living.

### 3.2 Changes in Care Demands, Resources, Perceptions, and Caregiver Wellbeing

Table 2 presents the changes in care demands, resources, perceptions, and caregivers well‐being. Over the 2 years, the care burden of the participants remained stable, and quality of life slightly increased despite an increase in care demands (decline in ADLs of older adults). They spent less time on the caregiving daily, along with an increase in the size of caregiving family, more use of caregiver support services, and improvement in the quality of the relationship with the older adult.

**TABLE 2:** Changes in care demands, family resources, perceptions, and caregiver wellbeing over 2 years (*n* = 93).

Domains (possible range)	Baseline	2‐year follow‐up	Paired *t*/McNemar test
	*M* (SD)/*n* (%)		*p*
**Context of care**			
Daily hours of caregiving	14.2 (6.8)	11.0 (5.1)	< 0.001***
**Care demands**			
Barthel Index for ADLs (0–100)	60.1 (29.7)	48.9 (29.9)	< 0.001***
Lawton IADLs (0–18)	2.80 (3.51)	2.49 (3.72)	0.247
Additional caregiving role	12 (13%)	8 (9%)	0.344
Stopped providing care	—	7 (8%)	
Stable	—	83 (89%)	
Newly started	—	3 (3%)	
**Resources**			
Size of caregiving family	1.56 (0.76)	2.01 (1.05)	< 0.001***
Use of live‐in domestic helper	25 (27%)	29 (31%)	0.344
Stop hiring	—	3 (3%)	
Stable	—	83 (89%)	
Newly hired	—	7 (8%)	
Use of caregiver support service	11 (12%)	30 (32%)	< 0.001***
Stop using	—	5 (%)	
Stable	—	64 (69%)	
Newly used	—	24 (26%)	
**Perceptions**			
Quality of relationship with CR (1–5)	3.74 (0.75)	3.91 (0.89)	0.048*
Satisfaction with family support (1–5)	3.25 (0.83)	3.31 (1.06)	551
**Caregiver wellbeing**			
Zarit Burden Interview (0–88)	31.0 (15.0)	29.6 (16.4)	0.441
EUROHIS‐QoL 8‐item index (1–5)	3.11 (0.50)	3.22 (0.57)	0.045*

*Note:* *p < 0.05; ***p < 0.001.

Abbreviations: ADLs, Activities of daily living; CG, Caregiver; CR, Care recipient; EUROHIS‐QoL, European Health Interview Survey – Quality of Life; IADLs, Instrumental activities of daily living.

### 3.3 Association of Care Demands, Resources, and Perceptions With Wellbeing at Baseline

Results of the regression analyses on care burden and quality of life at baseline were presented in Table 3. A higher level of care burden was associated with female gender, higher educational level, longer hours of caregiving, additional caregiving roles, poorer quality of relationship with CR, and lower satisfaction with family support. Better quality of life was associated with a higher level of ADLs of the older adults, without an additional caregiving role, better quality of relationship with CR, and higher satisfaction with family support. Factors of resources were not associated with either care burden or quality of life at baseline. Among the family‐level factors, additional caregiving role and satisfaction with family support, but not size of caregiving family, were associated with both indicators of caregiver well‐being.

**TABLE 3:** Predicting caregiver well‐being by demands, resources, and perceptions at baseline (*N* = 358).

Factor	Care burden			Quality of life		
	*B* (SE)	*β*	*p*	*B* (SE)	*β*	*p*
**Context of care**						
CG gender (female)	−3.74 (1.75)	−0.11	0.033*	0.05 (0.06)	0.04	0.435
CG education level	2.33 (0.71)	0.17	0.001**	0.00 (0.02)	0.00	0.953
Cohabitating with CR	0.85 (2.40)	0.02	0.722	−0.04 (0.08)	−0.03	0.576
Daily hours of caregiving	0.34 (0.12)	0.15	0.006**	0.00 (0.00)	0.04	0.430
Monthly household income per head	0.00 (0.00)	0.06	0.197	0.00 (0.00)	0.00	0.981
**Care demands**						
ADLs	−0.03 (0.03)	−0.05	0.418	0.00 (0.00)	0.15	0.014*
IADLs	−0.40 (0.24)	−0.09	0.105	0.00 (0.01)	0.01	0.808
Additional caregiving role	4.48 (2.11)	0.10	0.034*	−0.20 (0.07)	−0.15	0.003**
**Resources**						
Size of caregiving family	0.26 (0.94)	0.01	0.782	0.01 (0.03)	0.01	0.811
Use of live‐in domestic helper	1.66 (1.91)	0.05	0.386	0.03 (0.06)	0.02	0.693
Use of caregiver support service	2.17 (2.46)	0.04	0.378	0.13 (0.08)	0.08	0.102
**Perceptions**						
Quality of relationship with CR	−3.55 (1.06)	−0.17	< 0.001***	0.13 (0.04)	0.20	< 0.001***
Satisfaction with family support	−5.72 (1.00)	−0.29	< 0.001***	0.19 (0.03)	0.29	< 0.001***

*Note:* Reference category is shown in parentheses. Care burden model: Δ*R* ^2^ = 0.148 (Δ*F* (8341) = 8.211, *p* < 0.001) after controlling for context of care. Quality of life model: Δ*R* ^2^ = 0.181 (Δ*F* (8338) = 9.512, *p* < 0.001) after controlling for context of care. *p < 0.05; **p < 0.01; ***p < 0.001.

Abbreviations: ADLs, Activities of daily living; CG, Caregiver; CR, Care recipient; IADLs, Instrumental activities of daily living.

### 3.4 Predicting Wellbeing by Care Demands, Resources, Perceptions, and Their Changes

Table 4 summarizes the results of the regression models for predicting care burden and quality of life. When all the theory‐driven predictors were entered into the model (model 1), only seven factors showed at least a small part correlation (*r* > 0.01) for both care burden and quality of life. These factors were then entered into model 2. Predictors of higher care burden were longer caregiving hours at baseline, an increase in hours of caregiving, a new additional caregiving role, a decrease in the size of the caregiving family, and not using a domestic helper at baseline. Predictors of better quality of life at follow‐up were the use of a domestic helper at baseline and an increase in satisfaction with family support. Among the factors of change, only the changes in caregiving hours, additional caregiving role, and size of the caregiving family predicted care burden, and only the change in satisfaction with family support predicted quality of life.

**TABLE 4:** Predicting caregiver wellbeing by demands, resources, and perceptions in 2 years (*n* = 93).

Factor	Care burden						Quality of life					
	Model 1			Model 2			Model 1			Model 2		
	*B* (SE)	*β*	*p*	*B* (SE)	*β*	*p*	*B* (SE)	*β*	*p*	*B* (SE)	*β*	*p*
Baseline care burden/quality of life	0.29 (0.12)	0.26	0.018*	0.34 (0.09)	0.32	< 0.001***	0.54 (0.12)	0.48	< 0.001***	0.64 (0.1)	0.57	< 0.001***
**Context of care**												
CG gender (female)	−2.24 (3.44)	−0.06	0.517	—	—	—	0.11 (0.12)	0.08	0.390	—	—	—
CG education level	0.07 (1.52)	0.01	0.964	—	—	—	0.03 (0.06)	0.06	0.617	—	—	—
Cohabitating with CR	−1.26 (4.74)	−0.03	0.791	—	—	—	0.12 (0.17)	0.07	0.496	—	—	—
Daily hours of caregiving, baseline	0.93 (0.34)	0.39	0.008**	0.94 (0.26)	0.39	< 0.001***	−0.02 (0.01)	−0.20	0.188	−0.01 (0.01)	−0.15	0.212
Daily hours of caregiving, change	0.46 (0.34)	0.18	0.180	0.57 (0.27)	0.22	0.042*	−0.02 (0.01)	−0.21	0.138	−0.02 (0.01)	−0.22	0.072
Monthly household income per head	0.00 (0.00)	−0.04	0.673	—	—	—	0.00 (0.00)	0.02	0.855	—	—	—
**Care demands**												
ADLs, baseline	−0.02 (0.07)	−0.04	0.726	—	—	—	0.00 (0.00)	0.00	0.982	—	—	—
ADLs, change	−0.03 (0.08)	−0.04	0.698	—	—	—	0.00 (0.00)	−0.13	0.279	—	—	—
IADLs, baseline	0.30 (0.56)	0.06	0.595	—	—	—	−0.01 (0.02)	−0.06	0.645	—	—	—
IADLs, change	0.28 (0.69)	0.04	0.689	—	—	—	0.00 (0.03)	−0.01	0.964	—	—	—
Additional caregiving role, baseline	−1.97 (6.92)	−0.04	0.776	—	—	—	−0.08 (0.25)	−0.05	0.743	—	—	—
Additional caregiving role, stopped	−9.82 (8.77)	−0.16	0.267	−12.88 (4.60)	−0.21	0.006**	0.36 (0.32)	0.17	0.262	—	—	—
Additional caregiving role, newly started	17.9 (8.94)	0.19	0.049*	20.11 (7.09)	0.22	0.006**	0.03 (0.32)	0.01	0.931	—	—	—
**Resources**												
Size of caregiving family, baseline	0.37 (2.19)	0.02	0.866	—	—	—	0.00 (0.08)	0.00	0.971	—	—	—
Size of caregiving family, change	−7.12 (1.67)	−0.45	< 0.001***	−7.47 (1.22)	−0.47	< 0.001***	0.01 (0.06)	0.02	0.882	—	—	—
Use of live‐in domestic helper, baseline	−10.31 (3.78)	−0.28	0.008**	−9.62 (2.84)	−0.26	0.001**	0.21 (0.14)	0.17	0.129	0.23 (0.11)	0.18	0.040*
Use of live‐in domestic helper, stopped hiring	6.27 (8.76)	0.07	0.477	—	—	—	−0.08 (0.32)	−0.03	0.803	—	—	—
Use of live‐in domestic helper, newly hired	−1.22 (5.85)	−0.02	0.835	—	—	—	0.22 (0.21)	0.10	0.293	—	—	—
Use of caregiver support service, baseline	−1.23 (6.01)	−0.02	0.839	—	—	—	0.33 (0.22)	0.19	0.136	0.34 (0.20)	0.20	0.082
Use of caregiver support service, stop using	1.3 (9.21)	0.02	0.888	—	—	—	−0.39 (0.34)	−0.16	0.261	−0.48 (0.28)	−0.19	0.097
Use of caregiver support service, new user	−2.08 (3.53)	−0.06	0.558	—	—	—	0.23 (0.13)	0.18	0.085	0.20 (0.11)	0.16	0.065
**Perceptions**												
Quality of relationship with CR, baseline	−1.52 (2.37)	−0.07	0.524	—	—	—	0.08 (0.09)	0.10	0.381	—	—	—
Quality of relationship with CR, change	−1.35 (2.01)	−0.07	0.504	—	—	—	0.07 (0.08)	0.10	0.363	—	—	—
Satisfaction with family support, baseline	−3.98 (2.37)	−0.20	0.098	−2.71 (1.55)	−0.14	0.084	0.06 (0.08)	0.09	0.454	—	—	—
Satisfaction with family support, change	−2.17 (1.72)	−0.14	0.210	—	—	—	0.12 (0.06)	0.23	0.050	0.11 (0.05)	0.19	0.022*

*Note:* Reference category is shown in parentheses. Care burden model 1: Δ*R* ^2^ = 0.358 (Δ*F* (19,66) = 3.144, *p* < 0.001) after controlling for context of care and baseline care burden; Care burden model 2: Δ*R* ^2^ = 0.414 (Δ*F* (7,84) = 11.377, *p* < 0.001) after controlling for baseline care burden; Quality of life model 1: Δ*R* ^2^ = 0.202 (Δ*F* (19,66) = 1.583, *p* = 0.087) after controlling for context of care and baseline quality of life; Quality of life model 2: Δ*R* ^2^ = 0.148 (Δ*F* (7,84) = 3.296, *p* = 0.004) after controlling for baseline quality of life. *p < 0.05; **p < 0.01; ***p < 0.001.

Abbreviations: ADLs, Activities of daily living; CG, Caregiver; CR, Care recipient; IADLs, Instrumental activities of daily living.

## 4 Discussion

To our knowledge, this is the first study to apply the family stress theory to understand the well‐being of caregivers of older adults and comprehensively include factors of demands, resources, and perceptions in the investigation. For care demands, having an additional caregiving role was found to be associated with a higher care burden and poorer quality of life at baseline, and its changes, including stopping and starting to care for another family member, predicted lower and higher care burden, respectively. Higher functional ability (ADLs) of the older adults was only associated with better quality of life at baseline. For resources, an increase in the size of the caregiving family predicted lower care burden, and the use of a domestic helper predicted both a lower care burden and a better quality of life. For perceptions, we found that both better quality of relationship with CR and higher satisfaction with family support were associated with lower care burden and better quality of life at baseline, and an increase in satisfaction with family support predicted better quality of life.

Family stress theory provided a perspective for understanding how primary caregivers, as part of a family, maintained their well‐being during the caregiving journey. We conceptualized family caregiving as an enduring stressful event in which the family as a unit responded by mobilizing its resources to maintain balanced family functioning. In this sample, there was a significant increase in care demands in 2 years (decline in ADLs of the older adults). The families adaptively responded by involving more family members in caregiving, maintaining the use of domestic helpers, and using more formal caregiver support services, with their perception about the care demand (quality of relationship with CR) improving and perception about family resources (satisfaction with family support) remaining stable. It resulted in a stable level of caregiver well‐being over the 2 years. While we emphasize that a family should be treated as a unit in the context of caregiving, subjected to the data available, we only examined the well‐being of the primary caregiver in the present study. Further research should expand the investigation into the outcomes of the whole family, such as well‐being, adaptation, family functioning, and family quality of life (Turnbull et al. 2007).

One of the strengths of applying family stress theory is to expand our understanding of the demand faced by the caregiver beyond the caregiving role and examine the impact of competing roles on caregivers' well‐being. Even only for caregiving, a caregiver could have multiple caregiving roles at the same time. In our sample, we captured whether the caregivers had additional caregiving roles (17%) and found that it was associated with higher care burden and poorer quality of life at baseline, and its changes predicted care burden at follow‐up. Previous studies on caregivers of adults with intellectual disabilities similarly showed that additional caregiving roles, also known as compound caregiving, were associated with increased desire to place the care recipients into residential care (Perkins and Haley 2010). These results altogether suggested that family and caregiver support service providers should identify and target caregivers with multiple caregiving roles. Further caregiving research may expand into other family roles and examine their impact on caregivers' well‐being.

Separating the resources and perceptions conceptually is another strength of the family stress theory. Although it was to our surprise that all the factors of resources (size of caregiving family, use of domestic helper, and use of caregiver support service) were not associated with caregiver well‐being at baseline, the associations between perceptions (quality of relationship with CR and satisfaction with family support) and well‐being echoed with the literature and further confirmed that perceptions matter. Previous studies show that caregivers who perceived a lack of choice in taking on the caregiving role (Schulz et al. 2012) and felt trapped by the role (Campbell et al. 2008) reported higher emotional stress and care burden. Brodaty and Donkin (2009) similarly summarized that perceived support from the family, not only the actual support, and satisfaction with the support network predicted the well‐being of caregivers of people with dementia. While interventions on care demands (e.g., rehabilitation programs for improving/maintaining functional abilities of older adults) and family resources (e.g., policies supporting caregiver‐friendly employment, connecting families with community resources) should continue, perceptions about the caregiving role and family support are also potential intervention targets crucial to caregiver wellbeing.

With two time points of data, we attempted to model the pile‐up of stressors and the adaptive responses of a family along the caregiving process for predicting caregiver well‐being and found that the change in additional caregiving role (care demand) and the change in size of the caregiving family (resource), but not their baseline status/value, were predictive for care burden at follow‐up. Our results were in line with the Family Adjustment and Adaptation Response model (Patterson 1988) and supported that it was the imbalance between demand and resources that was challenging to family well‐being. Further research with more time points and larger samples to assess the longitudinal changes in care demands and resources and their contributions to caregiver well‐being is warranted.

Among the factors of family resources, the use of a live‐in domestic helper was predictive of both lower care burden and better quality of life at follow‐up. While earlier local studies found that domestic helpers mitigate the impact of care demands on caregiver distress (Chong et al. 2014, 2017), we identified similar benefits for caregiver well‐being. It is noteworthy that, despite focusing on the lower‐income group (below 75% of the local median monthly domestic household income), the prevalence of the use of domestic helpers in our sample (29% among low‐income caregivers of older adults awaiting long‐term care services in 2015) was slightly higher than that in the overall income sample (21% among older adults with long‐term care needs in 2021 census data; Census and Statistics Department of HKSAR Government 2023). This is probably because the caregiver allowance program was targeting those not yet supported by formal care services, leading to a greater need for domestic help. Furthermore, the use of domestic helper remained stable over the 2 years of receiving the caregiver allowance, which could nearly subsidize half the cost. These findings altogether suggest that income was not a determining factor for hiring domestic helpers in these families. Even having financial struggles or extra financial support, family caregivers prioritized the care needs of older adults and available support when deciding on hiring help, which is very much in line with the filial behaviors observed in Hong Kong (Lum et al. 2016b; Yeh et al. 2013). Further research using income subgroup analysis is needed to explore how low income affects families' access to such extra help and its consequences on caregiver well‐being.

Another resource factor predicting caregiver well‐being is the change in the size of the caregiving family, which has been less studied previously. In this study, the number of people involved in caregiving was defined broadly to include ADL/IADL support, emotional support, and major decision‐making. The findings suggest that interventions that promote family involvement in any type of care, beyond just direct care, could have a positive impact on caregiving wellbeing. This aligns with the shift in filial behavior, where symbolic or financial support but not direct care provision is increasingly observed (Cheung and Kwan 2009; Ting 2009).

Lastly, regarding the context of care, family income did not show an association with caregiver well‐being in the current study. Yet, previous studies reported that lower income (Chiao, Wu, and Hsiao 2015) and higher financial stress (Adelman et al. 2014) are risk factors for high care burden. This inconsistency is likely because the current sample was entirely recruited from low‐income families (with household income below 75% of the local median monthly domestic household income), which may not fully represent the broader population. Moreover, unlike previous studies, we included income, the use of domestic helpers, and the involvement of multiple family caregivers in our predictive model. The findings suggest that human resources (domestic helpers and size of the caregiving family) are better predictors of caregiver well‐being than financial resources.

### 4.1 Limitations

There are several limitations in this study. First, due to the nature of the study (secondary analysis using policy evaluation research data), the sample size in the theory‐driven longitudinal model (model 1) was insufficient to support a valid regression analysis that required at least 10 observations per variable. Although we attempted to address the issue by adopting a combined theory‐ and data‐driven approach for selecting predictors in the final model, results should be interpreted with caution, and further research is needed to replicate findings from this study. Second, compared with those in the follow‐up subsample, caregivers who did not participate were younger and more likely to be employed. Previous studies had suggested that younger caregivers with competing commitments (e.g., employment) often report higher levels of stress (Pinquart and Sörensen 2003). The current study might have excluded caregivers with a higher level of care burden at the follow‐up. Nevertheless, the completers and the dropouts did not differ in their level of burden at baseline. Third, another limitation with the secondary data is that the variables we selected may not best represent the constructs in the family stress theory, in particular, perceptions about care demands and family resources. The current way of defining care demands resources, and perceptions can be considered a proof of concept of applying family stress theory in understanding caregiver well‐being. More carefully designed variables will be needed in future studies.

### 4.2 Implications

This study provided initial evidence of the applicability of family stress theory in understanding the caregiving process for older adults with care needs, with insight into the importance of competing family roles, change in family resources, and perceptions on caregiver well‐being. As societies are aging rapidly with decreasing availability of unpaid caregivers, viable solutions to support family care are urgently needed. To prevent caregiver burnout and maintain their quality of life, practitioners should pay attention to caregivers with multiple caregiving roles and develop routines to regularly monitor the number of family members involved in caregiving. This could be implemented under the existing caregiver allowance program, which requires caregivers to meet monthly with a designated social worker in their district. Specifically, we suggest including questions about any additional caregiving roles and the number of family members involved in caregiving during these regular meetings. If the social worker identifies a new caregiving role or a reduction in family involvement (indicating a potential imbalance between care demands and resources), the caregiver should be referred for further assessment of caregiver stress and overall family well‐being. Moreover, perceptions, including how caregivers view their relationships with older adults and the support they receive from other family members, may be potential intervention targets for improving caregiver well‐being. Caregiver support services, such as caregiving skill training, counseling, and mutual support groups, are available at all publicly funded community care service units throughout Hong Kong (Choy et al. 2022). However, most existing services focus on direct care provision, stress management, and relationships with older adults. There is currently a lack of psychosocial interventions that address how primary caregivers perceive support from other family members and how multiple caregivers share the caregiving responsibilities. Such individual‐ or family‐centered interventions need to be developed as a potential alternative to enhance our overall caregiver support services.

Our findings also shed light on how the caregiver allowance program affects caregiver well‐being and provide recommendations for future implementation, particularly in the Hong Kong context. The two main components of this allowance program are the monthly cash allowance and meetings with a designated social worker. Regarding the cash allowance, our findings suggest that factors such as additional caregiving roles, the use of domestic helpers, the quality of relationship with CR, the involvement of more family members, and satisfaction with family support are crucial for caregiver well‐being. Therefore, the current non‐restrictive use of the allowance should continue. This flexibility allows caregivers to spend the allowance on various purposes, such as supplementing caregiving expenses incurred by any family members with care needs, hiring a domestic helper, leisure activities with the CR, or even covering the living expenses of another family caregiver in exchange for their involvement. As for the monthly meetings, as we recommended earlier, the designated social worker should regularly assess whether there are changes in the number of family members requiring care from the caregiver and those involved in care provision. This will help identify the caregivers at higher risk of poor well‐being.

## Conflicts of Interest

The authors declare no conflicts of interest.
